# Identification of isothiazolones analogues as potent bactericidal agents against antibiotic resistant CRE and MRSA strains

**DOI:** 10.1186/s13065-023-01100-3

**Published:** 2023-12-16

**Authors:** Wenbin Jin, Chen Xu, Ning Dong, Kaichao Chen, Die Zhang, Jinhua Ning, Yunbing Li, Guangfen Zhang, Jin Ke, Anguo Hou, Linyun Chen, Sheng Chen, Kin-Fai Chan

**Affiliations:** 1 Key Laboratory of External Drug Delivery System and Preparation Technology in Universities of Yunnan and Faculty of Chinese Materia Medica, Yunnan University of Chinese Medicine, Kunming, Yunnan China; 2 State Key Laboratory of Chemical Biology and Drug Discovery and Department of Applied Biology and Chemical Technology, The Hong Kong Polytechnic University, Hung Hom, Kowloon, Hong Kong China; 3 School of Medicine, Jiangsu University, Zhenjiang, Jiangsu China; 4 Department of Food Science and Nutrition, The Hong Kong Polytechnic University, Hung Hom, Kowloon, Hong Kong China; 5 Department of Medical Microbiology, School of Biology and Basic Medical Sciences, Suzhou Medical College of Soochow University, Suzhou, China

**Keywords:** Bactericidal agents, Isothiazolones analogues, Antimicrobial resistance, MRSA, CRE

## Abstract

Carbapenem-resistant Enterobacterales (CRE) has emerged as a worldwide spread nosocomial superbug exhibiting antimicrobial resistance (AMR) to all current antibiotics, leaving limited options for treating its infection. To discovery novel antibiotics against CRE, we designed and synthesized a series of 14 isothiazol-3(2H)-one analogues subjected to antibacterial activity evaluation against *Escherichia coli* (*E. coli*) BL21 (NDM-1) and clinical strain *E. coli* HN88 for investigating their structure–activity relationships (SAR). The results suggested that 5-chloroisothiazolone core with an N-(4-chlorophenyl) substitution **5a** was the most potent antibacterial activity against the *E. coli* BL21 (NDM-1) with MIC value of less than 0.032 μg/mL, which was at least 8000-fold higher than the positive control Meropenem (MRM). It also displayed 2048-fold potent than the positive control MRM against *E. coli* HN88. Additionally, SAR analysis supported the conclusion that compounds with a chloro-group substituted on the 5-position of the heterocyclic ring was much more potent than other positions. The board spectrum analysis suggested that compound **5a** showed a promising antimicrobial activity on MRSA and CRE pathogens. Meanwhile, cytotoxicity study of compound **5a** suggested that it had a therapeutic index value of 875, suggesting future therapeutic potential. In vivo efficacy study declared that compound **5a** could also protect the BALB/c mice against American type culture collection (ATCC) 43,300. Further screening of our compounds against a collection of CRE strains isolated from patients indicated that compound **5 g** displayed much stronger antibacterial activity compared with MRM. In conclusion, our studies indicated that isothiazolones analogues could be potent bactericidal agents against CRE and MRSA pathogens.

**Supplementary Information:**

The online version contains supplementary material available at 10.1186/s13065-023-01100-3.

## Introduction

**Fig. 1:**
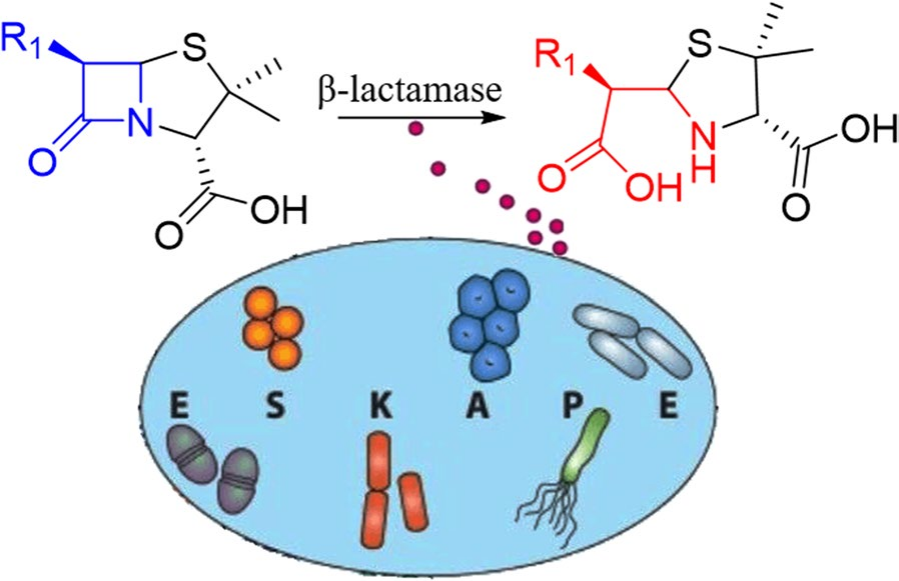
Antimicrobial resistance: β-lactamase modification (ESKAPE referred to *Enterococcus faecium, Staphylococcus aureus, Klebsiella pneumoniae, Acinetobacter baumannii, Pseudomonas aeruginosa* and *Enterobacter spp.*)

The serendipitous discovery of penicillin made by the Nobel laureate Alexander Fleming inaugurated the modern era of antibiotic utilization [1]. Since then lots of beta-lactam antibiotics, containing a beta-lactam ring as the scaffold, have been obtained. The beta-lactam antibiotics such as penicillins, cephalosporins, carbapenems and monobactams have been a cornerstone to treat infections caused by Gram-negative bacterial pathogen due to their efficacy and low toxicity to humans [2]. To date, more than 50% percent of antibiotics in clinical use belonged to β-lactam [3, 4]. Unfortunately, the Center for Disease Control (CDC) declared that 50% of antibiotics prescriptions in the hospital setting and nursing homes were either inappropriate or unnecessary [5, 6]. Eventually, the abuse of the antibiotics eventually led to the emergence of bacterial resistance. The expression of beta-lactamases rendering resistance to beta-lactam antibiotics by breaking the beta-lactam ring that is essential for the bactericidal activity posed a serious threat to human health (Fig. 1) [7].

**Fig. 2:**
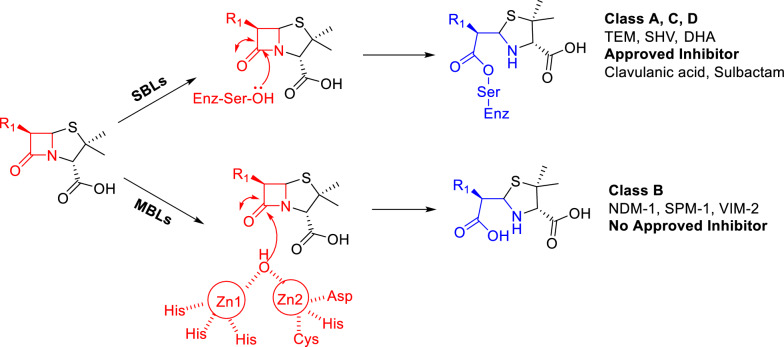
Classification and Mechanism of beta-lactamases

Based on the different amino acid sequences and functional mechanisms, beta-lactamases can be classified into four classes: Three serine-dependent enzyme classes A, C, and D known as serine-*β*-lactamases (SBLs) employed an active site serine to nucleophilically attack on the *β*-lactam carbonyl [8]. Widely accepted inhibitors of SBLs have been used in clinical such as sulbactam, tazobactam, and clavulanic acid [9]. One metal-dependent enzyme class B called metallo-*β*-lactamases (MBLs) adopted zinc-bound hydroxyl to nucleophilically attack the carbonyl group of β-lactam (Fig. 2) [10]. Clinically proven inhibitors of MBLs are still unavailable up to date [11]. Still worse, MBLs are not only encoded by horizontally transferable plasmids but also associated commonly with genes encoding for other antibiotic resistance determinants, conferring Carbapenem-resistant Enterobacterales (CRE) ‘‘superbugs’’ which exhibits antimicrobial resistance (AMR) to nearly all current antibiotics [12]. What’s more, the CDC has manifested that the golden age of carbapenem set to end due to the “ESKAPE superbugs” which confer infections that require development of new effective antibiotics for treatment [13]. In particular, the rapid worldwide dissemination of NDM-1-producing“superbugs” further emphasizes the significant role of this type of carbapenemases in conferring antimicrobial resistance.

Recently the tremendous effort to discover potential NDM-1 inhibitors have been made [14]. Numerous NDM-1 inhibitors have been reported, but none of them have been approved by FDA [15]. Only cyclic boronate derivatives including VNRX-5133 and QPX7728 entered phase III and phase I clinical trial, respectively [16, 17]. Besides the approach to discover NDM-1 inhibitors to treat the currently untreatable infections by CRE, an alternative reliable strategy is to find a novel antibiotic with the ability to fight antibiotic resistance [18]. During the period of searching for the NDM-1 inhibitors, we have also disclosed a novel series of potent antibiotics referred to isothiazolones analogues [7, 19, 20]. The isothiazolone analogues reported before have displayed broad-spectrum antibacterial activity against Gram-negative and Gram-positive strains [21, 22]. In this paper, we further expanded the scope of compounds and determined whether their bactericidal effects against non-resistance pathogens could also be replicated in a panel of CRE and MRSA and investigated structure-antibacterial activity relationships of isothiazolones against inducible Carbapenem-resistant *E. coli* BL21 carrying Pet28-blaNDM-1 and clinical isolated strain *E. coli* HN88 carrying blaNDM-1.

## Results and discussion

### Chemistry

**Fig. 3:**
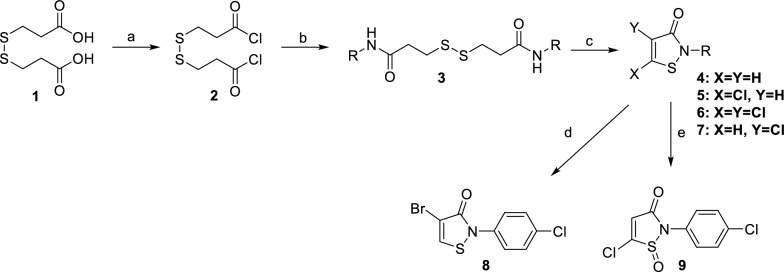
Synthesis route of designed compounds. Regents and conditions: **a** SOCl_2_, DMF, DCM, reflux, 1 h; **b** corresponding amine, Et_3_N, DCM, ice bath to r. t, 18 h; **c** SO_2_Cl_2_, DCM, ice bath to r. t, 18 h; **d** Br_2_, EA, ice bath to r. t, overnight; **e** mCPBA, CH_2_Cl_2_, r.t, overnight

The synthetic route for the preparation of a collection of N-functionalized isothiazolones derivatives reported previously was outlined in Fig. 3 [23]. Starting from commercially available 3,3'-disulfanediyldipropionic acid **1**, treatment with thionyl chloride in Dichloromethane (DCM) solvent with *N,N*-Dimethylformamide (DMF) as catalyst afforded the acyl chloride **2**, which was further reacted with excess corresponding amine gave the dithiodipropionamides **3**, followed by subsequent chlorine-assisted cyclization of the diamide with sulfuryl chloride using DCM as a solvent in an ice bath afforded the desired isothiazol-3(2H)-ones **4–6** [24]. Upon modification of the reagent stoichiometry, various products could be obtained as it was suggesting that the 5-unsubstituted analogues **4** were the single products with moderate yields when the ratio of diamide to sulfuryl chloride was 1:1, the 5-chloroisothiazolone derivatives **5** were the predominant products with 4-chloroisothiazolone derivatives **7** as the side products while with the relevant ratio of 1:3, and while with the ratio of 1:5 afforded the 4,5-dichloroisothiazolone derivatives **6** as the major product. Further bromination of compound **4a** gave 4-bromoisothiazolone derivative **8**. Oxidation of 5-chloroisothiazolone compound **5a** with 3-chloroperoxybenzoic acid (m-CPBA) in an ice bath gave the 5-chloroisothiazol-3(2H)-one-1-oxide **9** in a low yield.

### In vitro bactericidal screening

#### Cell-based bactericidal screen using *E. coli* BL21 (NDM-1) and clinical strain *E. coli* HN88 identified compound 5a as a promising antibiotic against superbugs.

MRM-resistant *E. coli* BL21 (NDM-1) carrying only an Isopropyl-*β*-D-thiogalactopyranoside (IPTG)-inducible plasmid pET28b-bla NDM-1 was produced from a parental *E. coli* BL21 strain without producing NDM-1. *E. coli* HN88 carrying blaNDM-1 was collected from clinical patients. All cell-based bactericidal screen using *E. coli* BL21 (NDM-1) and clinical strain *E. coli* HN88 were conducted according to the Clinical and Laboratory Standards Institute (CLSI) guidelines. The minimum inhibition concentration (MIC) of MRM towards these antibiotic resistance strains of *E. coli* BL21 (NDM-1) and *E. coli* HN88 were found to be greater than 256 μg/mL (Table 1, entry 1), which was at least 2056-fold higher than the parental *E. coli* BL21 with antibiotic susceptibility (MIC of MRM = 0.125 μg/mL).

**Table 1:** MIC screening, cLogP and tPSA of isothiazol-3(2H)-ones and their combination with MRM at ratio of 1:1 against *E. coli* BL21 (NDM-1) strain and clinically isolated *E. coli* HN88 (NDM-1) strain 
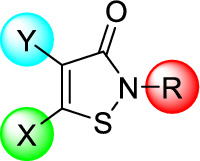

Entry	Compound				BL21		FICI	HN88		FICI	ClogP	TpsA
					Cpd alone	MRM: Cpd		Cpd alone	MRM: Cpd			
1	MRM	N.A	N.A	N.A	≥ 256	N.A	N.A	≥ 256	N.A	N.A	N.A	N.A
2	4a		H	H	2	1	0.503906	8	4	0.515625	2.472	20.31
3	4b		H	H	≥ 256	128	1	≥ 256	128	1	0.894	63.68
4	4c		H	H	≥ 256	≥ 256	2	≥ 256	128	1	2.171	20.31
5	5a		H	Cl	≤ 0.032	0.125	3.906738	2	2	1.007813	3.185	20.31
6	5b		H	Cl	≥ 256	128	1	≥ 256	128	1	1.607	63.68
7	5c		H	Cl	8	≤ 4	0.515625	32	16	0.5625	3.0238	29.54
8	5d		H	Cl	≥ 256	128	1	≥ 256	128	1	2.634	20.31
9	5e		H	Cl	≥ 256	64	0.5	≥ 256	128	1	1.575	66.81
10	5f	C_8_H_17_	H	Cl	4	≤ 1	0.253906	4	8	2.03125	− 4.203	20.31
11	5 g		H	Cl	0.064	0.125	1.9536	4	4	1.0156	0.703	20.31
12	6		Cl	Cl	≥ 256	32	0.25	≥ 256	≥ 256	2	3.778	20.31
13	7		Cl	H	8 or 16	2	0.257813	16	16	1.0625	3.185	20.31
14	8		Br	H	4	4	1.015625	32	16	0.5625	3.335	3.335
15	9		H	Cl	≥ 128	≥ 128	1.5	≥ 128	≥ 128	1.5	− 0.273	29.1

MIC value were determined using the double dilution method in accordance with the CLSI guidelines by which bactericide effect was assessed by naked eye after overnight incubation. All MIC experiments were performed at least triplicate

#### Structure–activity relationship study of isothiazol-3(2H)-ones for bactericidal

A total of 14 isothiazol-3(2H)-one analogues were designed, synthesized and simultaneously subjected to assessment of bacterial-killing activity against *E. coli* BL21 (NDM-1) and clinical strain *E. coli* HN88 for investigating their structure–activity relationship. In this study, structural modification of these novel isothiazol-3(2H)-one analogues were mainly focused on its 4- and 5- substituents positions, as well as the N-position (Fig. 3). These antibacterial screen results were summarized as shown in Table 1. MRM was selected to act as positive control (**Entry 1, **Table 1).

In order to reveal the functional scaffold for the antibacterial activity of isothiazol-3(2H)-ones, several series isothiazolones with N-position groups different in hydrophobicity, group size, steric and electronic parameters were synthesized and evaluated of their antibiotic activity against the resistant strains. Most compounds showed ignored or no antibacterial effect with MIC ≥ 8 μg/mL except compound **4a**, **5a**, **5f**, **5g** and **8**, indicating that compounds with N-phenyl ring substituted with 4-chloro group would be more beneficial such as compound **4a** and **5a**. Next, by analysis of the MIC values of compound **4a, 5a, 6, 7** and **8** with the same N-phenyl ring substituted with 4-chloro group, we could draw the conclusion that 5-chloroisothiazolone core with an N-(4-chlorophenyl) substitution **5a** had the highest antibacterial activity among the corresponding C-5 unsubstituted analogues (compound **4a**, **7** and **8**) and C-4,5 dichloro-substituted analogue **6**. Furthermore, compound **5a** (**Entry 7, **Table 1) showed the most potent antibacterial activity against the *E. coli* BL21 (NDM-1) with MIC value of less than 0.032 μg/mL, which was at least 8000-fold higher than the positive control MRM. It was also referred to *E. coli* HN88, suggesting that 2048-fold higher than the positive control MRM. Nevertheless, compound **9** obtained by oxidation of **5a** (**Entry 18, **Table 1) showed MIC value ≥ 128 μg/mL, suggesting that 5-chloroisothiazolone core was indispensable pharmacophore for bactericidal effect. Besides, for the *E. coli* BL21 carrying blaNDM-1, the MIC value of compound **5a** was found to be ≤ 0.032 μg/mL, conferring a more than 62.5-fold reduction in *E. coli* HN88 MIC which was 2 μg/mL, suggesting that the clinical strain was more resistant than the experimental one. Therefore, we selected compound **5a** as the lead candidate for further investigation on bactericidal effects against a panel of clinical isolated strains carrying various MBLs.

#### Synergistic study of tested compounds with conventional antibiotic MRM.

The combinational usage of two bactericidal agents could boost the bacterial susceptibility and prevent the antimicrobial resistance [25, 26]. Therefore, we next determined whether the isothiazol-3(2H)-ones had synergistic effect with conventional antibiotic MRM by incubating the *E. coli* BL21 (NDM-1) strain and clinically isolated *E. coli* HN88 (NDM-1) strain in the presence of tested compounds and MRM at the ratio of 1:1. The synergistic effect was determined by the FIC index, which was calculated as FIC (cpd) + FIC (MRM), where FIC (cpd) was defined as the (MIC_combination_)/(MIC_cpd_) and FIC (MRM) was the ratio of MIC_combination_ to MIC_MRM_. The drug combination was considered synergy if FIC Index was ≤ 0.5, no interaction if 0.5 < FICI ≤ 4, and antagonism if FICI > 4. The results as shown in Table 1 suggested that nearly all of the synthetic isothiazol-3(2H)-ones derivatives had no synergistic effect with MRM against the aforementioned strains with exception of compound **5f**, **6** and **7** with FICI value of approximately 0.25 on the *E. coli* BL21 (NDM-1) strain. The FICI between compound **5a** and MRM were larger than 3.9 on the *E. coli* BL21 (NDM-1) strain, indicating this compound **5a** had antagonistic effect with MRM.

#### Spectrum of activity of compounds 4a, 5a and 5 g

In order to demonstrate the board spectrum of the most potent compounds **4a**, **5a** and **5 g**, several ATCC strains including MRSA were used in the antimicrobial susceptibility testing (Table 2). The results suggested that compound **5a** showed a promising antimicrobial activity on Gram-negative pathogen *E. coli* and Gram-positive pathogen *S. aureus*. Compound **5 g** could kill the Gram-positive pathogen *S. aureus* with MIC value of 2 µg/mL.

**Table 2:** MIC (µg/mL) screening of compounds **4a**, **5a** and **5g** against on ATCC strains

Organism	5a	4a	5 g
*S. aureus* 1717	2	4	2
*S. aureus* 1749	2	4	2
*S. aureus* 43,300	1	4	2
*E. coli* 29,425	0.125	4	_
*E. coli* 25,113	0.25	4	_

#### MIC screening of compound 5a against clinically isolated Gram-negative CRE strains

On the basis of the MIC screening results of isothiazol-3(2H)-ones against *E. coli* BL21 (NDM-1) strain and *E. coli* HN88 (NDM-1) strain, we further checked whether their bactericidal effects in the aforementioned screening strains could also be reproduced in our in-house collection of a panel of 5 Gram-negative CRE strains including *E. coli, C. freundii, E. cloacae, K. pneumoniae and M. morganii* strains which were clinically isolated from patients in the Second People’s Hospital of Jiaxing in Zhejiang Province. These CRE strains are all NDM-1 positive and highly resistant to MRM, exhibiting MICs of MRM ranging from 64 µg/mL to ≥ 128 µg/mL (Table 2). Apart from expressing NDM-1 enzyme, all of the tested strains with exception of M. morganii could also produce other additional β-lactamases such as CTX-M-3, CTX-M-14, SHV-12, TEM-1 and KPC-2 to confer antimicrobial resistance (AMR). Encouragingly, as illustrated in Table 3, four compound including **4a**, **5f**, **5 g** and **7** demonstrated promising antibacterial activity itself (MICs ≤ 32 µg/mL), exhibiting much stronger antibacterial activity against the aforementioned pathogens compared with the positive compound MRM and else compounds.

**Table 3:** MIC (µg/mL) screening of MRM and isothiazol-3(2H)-ones against clinically isolated CRE strains carrying NDM-1 and additional β-lactamases

Compound	*Escherichia coli06* (EC06)	*Citrobacter freundii17* (CF17)	*Enterobacter cloacae27* (ECL27)	*Klebsiella pneumoniae1*4 (KP14)	*Morganella morganii2*3 (MM23)
MRM	≥ 128	≥ 128	64	≥ 128	64
4a	8	16	16	16	16
4b	≥ 128	≥ 128	≥ 128	≥ 128	≥ 128
4c	≥ 128	≥ 128	≥ 128	≥ 128	≥ 128
5a	≥ 128	≥ 128	≥ 128	≥ 128	≥ 128
5b	≥ 128	≥ 128	≥ 128	≥ 128	≥ 128
5c	≥ 128	≥ 128	≥ 128	≥ 128	≥ 128
5d	≥ 128	≥ 128	≥ 128	≥ 128	≥ 128
5e	≥ 128	≥ 128	≥ 128	≥ 128	≥ 128
5f	16	16	16	8	16
5 g	≤ 2	≤ 2	≤ 2	≤ 2	≤ 2
6	≥ 128	≥ 128	≥ 128	≥ 128	≥ 128
7	16	≥ 128	32	32	8
8	≥ 128	≥ 128	≥ 128	32	≥ 128
9	≥ 128	≥ 128	≥ 128	≥ 128	≥ 128

Clinical strains usually produce NDM-1 enzyme and other additional β-lactamases whose genes carried in different strains are detailed shown respectively. EC06 consists of CTX-M-3, CTX-M-14, SHV-12. CF17 consists of SHV-12. ECL27 consists of CTX-M-3, CTX-M-14, TEM-1, SHV-12. KP14 consists of CTX-M-14, KPC-2, SHV-12. MM23 only has NDM-1. MIC values were determined using the double dilution method in accordance with the CLSI guidelines and previous reports by which inhibition of bacterial growth was assessed by naked eye after overnight incubation. The results were obtained from MIC experiments performed at least triplicate

#### Growth curve and time-killing assay of compound 5a against *S. aureus* 43,300

**Fig. 4:**
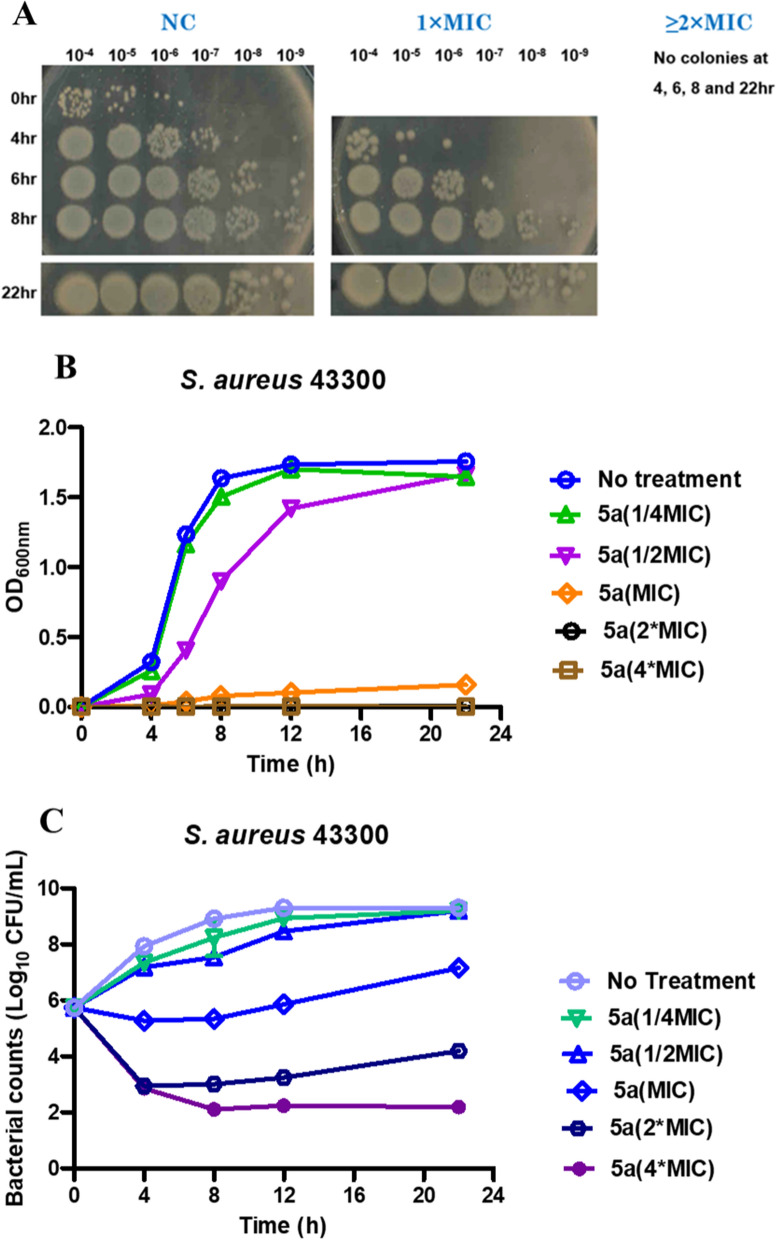
Anti-MRSA activity of compound **5a** in vitro. **A** Image of incubation of *S. aureus* with compound **5a** (**B**) Growth curve of compound **5a** against *S. aureus* 43,300. The X-axis shows the time (h), and the Y-axis represents the OD_600_. **C** Time-killing assay of compound **5a** against *S. aureus* 43,300. Data are mean ± SEM for three independent experiments

Time-killing assay used to unveil the dynamic interaction between antimicrobial agents and strains is the most useful method for determining the bactericidal effects, revealing a time-dependent and a concentration-dependent bactericidal effects [27, 28]. To determine the bactericidal effect of compound **5a**, the growth curve assay and the time-killing assay against *S. aureus* 43,300 were conducted against Gram-positive strain *S. aureus* **43,300**. The growth curve of the treatment of compound **5a** at MIC concentration displayed a gradual log reduction with OD_600_ value ranging from 0.159 to 0.001 within 4 h. Furthermore, incubating of *S. aureus* with compound **5a** at > twofold MIC suggested that no colonies formed at 4, 6, 8 and 22 h (Fig. 4A, B). In addition, the bacterial survival rates of *S. aureus* 43,300 exposed to compound **5a** at various concentrations ranging from 1/4 MIC to 4 MIC at different times (Fig. 4C) were measured. The aftermath suggested that any concentration of **5a** could not kill bacteria within 4 h, and only fourfold MIC of **5a** could kill most bacteria within 22 h.

#### Cytotoxicity studies of compounds against eukaryotic cells

**Fig. 5:**
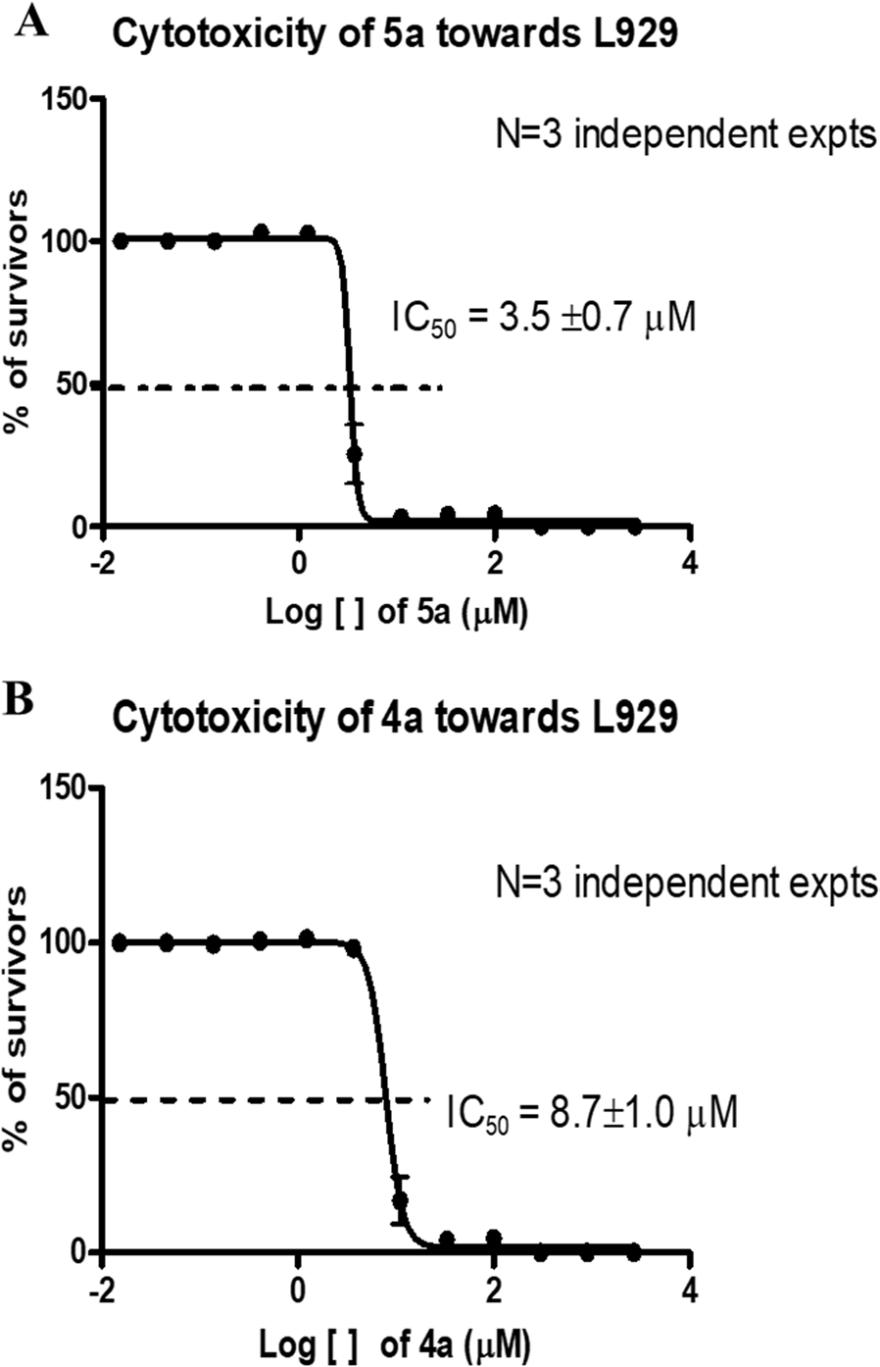
Cytotoxicity of compound **5a (A)** and **4a (B)** against L929 cell lines

Given that the cytotoxicity towards eukaryotic cells has been the greatest obstacle to the development of bactericidal, mouse peritoneal fibroblast L929 cell line was selected to verify the safety of the most potent compound **5a**. As shown in Fig. 5A, compound **5a** exhibited relatively low toxicity against L929 cell lines with IC50 value of 3.5 ± 0.7 μM, which is much higher than the MIC of 0.004 μM (1.0 μg/mL). Hence the therapeutic index calculated by IC_50_/MIC was 875, indicating that compound **5a** has a very broad therapeutic window. Moreover, cell morphology microscopic analysis suggested that no obvious morphological changes of L929 cells were observed after prolonged incubation with compound **5a** at the MIC, exhibiting negligible toxicity. In addition, compound **4a** also displayed relatively low toxicity against L929 cell lines with IC50 value of 8.7 ± 1.0 μM as shown in Fig. 5B, suggesting that this series of compounds could have sufficient margins of safety.

#### In vivo bactericidal action study of compound 5a

**Fig. 6:**
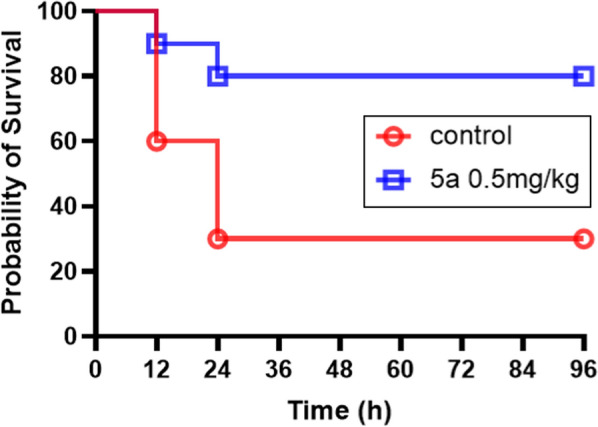
Kaplan–Meier survival curves of MRSA infected mouse following the injection of vehicle solution and compound **5a.** Data are the means of three independent experiments

To shed light on the potential clinical benefits of compound **5a**, in vivo efficacy was determined using a BALB/c mice infection model. The therapeutic abilities of compound **5a** at single doses of 0.5 mg/kg in drug monotherapy to protect the mice against a lethal dose infection of MRSA ATCC43300 (10^9^ CFU/mouse) through intravenous injection was evaluated. 70% mortality of mouse were observed for the control group of the vehicle consisting of 5% Cremophor EL, 5% ethanol and 90% saline after 24 h. Encouragingly, 0.5 mg/kg monotherapy of compound **5a** after 24 h resulted in 80% survival rate, suggesting that the excellent antibacterial ability of compound **5a** against MRSA ATCC43300 (Fig. 6). Compared to the treatment groups of the vehicle control group, it was found to be highly significant (p < 0.05). These results suggested that compound **5a** with strong potential should be worthy for further development in future.

## Conclusion

In this study, a total of 14 compounds isothiazol-3(2*H*)-one analogues were designed, synthesized and simultaneously subjected to assessment of bacterial-killing activity against *E. coli* BL21 (NDM-1) and clinical strain *E. coli* HN88. The SAR study suggested that 5-substituted chloride of the isothiazol-3(2*H*)-one enjoyed the priority. Compound **5a** displayed the most potent in vitro antibacterial activity against MRSA and CRE with the broad spectrum. In vivo study also suggested compound **5a** could enhance the survival rate in BALB/c mice infection model. Compound **5g** even could kill several clinical isolated CRE strains carrying various MBLs which was clinically isolated from patients. Altogether, our studies indicate that isothiazol-3(2*H*)-one derivatives provided a promising starting point to be further developed as broad-spectrum antibiotics against superbugs.

### Experimental section

#### General

Starting materials and reagents of commercial grade could be directly employed without further purification unless otherwise stated. All common reactions were visualized by TLC on aluminum sheets (Silica gel 60-F_254_, E. Merck) under UV light at 254 nm. Flash chromatography was performed on silica-gel 60 (200–300 mesh). ^1^H NMR (400 MHz) and ^13^C NMR (100 MHz) spectra were measured on a Bruker Advance-III spectrometer with TMS as an internal standard. Chemical shifts are expressed in δ (ppm) and coupling constants (*J*) in Hz (Additional file 1). High resolution MS spectra were measured using a QTOF-2 micromass Spectrometer by electron spray ionization.

To a well-stirred DCM solution (30 mL) of commercially available 3,3′-disulfanediyldipropanoic acid **1** (2.0 g, 9.52 mmol) was added dropwise thionyl chloride (2.48 g, 20.92 mmol) following dropping into DMF as the catalyst at 0 ◦C. The solution was heated under reflux for 12 h, the solvent was removed in vacuo and the crude residue was further reacted with corresponding amine (11.04 mmol) in DCM. After 6 h, the precipitate was filtered, washed with DCM, and dried in vacuo to yield the targeted diamide compound with sufficient purity for the next step. To a well-stirred solution of the diamide compound in DCM (25 mL) was added 1.0 equiv. of SO_2_Cl_2_ in an ice bath. After 2 h, the solution was poured into water and extracted with DCM. The organic layers were dried over anhydrous MgSO_4_, filtered and evaporated under reduced pressure. The resulting residue was purified by flash column chromatography on silica gel to afford the desired compound **4a–4c** as the major product.

#### 2-(4-chlorophenyl)isothiazol-3(2H)-one (4a)

This compound (yield 47.6%) was prepared from the 3,3'-disulfanediylbis(N-(4-chlorophenyl)propanamide) **3a** (0.43 g, 1.0 mmol) and sulfuryl chloride (0.13 g, 1.0 mmol) according to the general procedure described above. ^1^H NMR (400 MHz, CHLOROFORM-d) δ 8.15–8.25 (m, 1H), 7.48–7.56 (m, 2H), 7.35–7.42 (m, 2H), 6.29 (d, *J* = 6.85 Hz, 1H); ^13^C NMR (101 MHz, CHLOROFORM-d) δ 165.3, 146.6, 134.3, 133.4, 129.7, 125.9, 114.9. HRMS m/z calcd for [C_9_H_6_ClNOS + H] ^+^ 211.9931, found 211.9926.

#### 2-((4-methoxyphenyl)sulfonyl)isothiazol-3(2H)-one (4b)

This compound (yield 64.5%) was prepared from the 3,3'-disulfanediylbis(N-((4-methoxyphenyl)sulfonyl)propanamide) **3b** (0.55 g, 1.0 mmol) and sulfuryl chloride (0.13 g, 1.0 mmol) according to the general procedure described above. ^1^H NMR (400 MHz, CHLOROFORM-d) δ 8.59 (d, *J* = 4.89 Hz, 1H), 7.76–7.96 (m, 2H), 6.93–6.99 (m, *J* = 8.80 Hz, 2H), 6.88 (d, *J* = 4.89 Hz, 1H), 3.82 (s, 3H); ^13^C NMR (101 MHz, CHLOROFORM-d) δ 164.5, 159.6, 151.4, 130.9, 126.5, 115.2, 114.5, 55.8. HRMS m/z calcd for [C_9_H_6_ClNOS + Na] ^+^ 293.9865, found 293.9860.

#### 2-(4-bromobenzyl)isothiazol-3(2H)-one (4c)

This compound (yield 39.1%) was prepared from the 3,3'-disulfanediylbis(N-(4-bromobenzyl)propanamide) **3c** (0.55 g, 1.0 mmol) and sulfuryl chloride (0.13 g, 1.0 mmol) according to the general procedure described above. ^1^H NMR (400 MHz, CHLOROFORM-d) δ 8.42 (d, *J* = 3.91 Hz, 1H), 7.44—7.65 (m, *J* = 8.80 Hz, 2H), 7.21—7.44 (m, *J* = 8.80 Hz, 2H), 6.65 (d, *J* = 4.89 Hz, 1H), 5.40 (s, 2H); ^13^C NMR (101 MHz, CHLOROFORM-d) δ 169.2, 149.1, 135.7, 131.7, 129.8, 122.2, 111.9, 77.6, 77.3, 77.0, 69.7, 31.7, 22.8, 14.3. HRMS m/z calcd for [C_10_H_8_BrNOS + H] ^+^ 269.9510, found 269.9524.

To a well-stirred solution of the diamide compound in DCM (25 mL) and 4.5 equiv. Et_3_N was added 3.0 equiv. of SO_2_Cl_2_ in an ice bath. After 2 h, the solution was poured into water and extracted with DCM. The organic layers were dried over anhydrous MgSO_4_, filtered and evaporated under reduced pressure. The resulting residue was purified by flash column chromatography on silica gel to afford the desired compound **5a–5h** as the major product and **7** as the side compound.

#### 5-chloro-2-(4-chlorophenyl)isothiazol-3(2H)-one (5a)

This compound (yield 60.5%) was prepared from the 3,3'-disulfanediylbis(N-(4-chlorophenyl)propanamide) **3a** (0.43 g, 1.0 mmol) and sulfuryl chloride (0.39 g, 3.0 mmol) according to the general procedure described above. ^1^H NMR (400 MHz, CHLOROFORM-d) δ 7.45–7.54 (m, 2H), 7.37–7.45 (m, 2H), 6.37 (s, 1H); ^13^C NMR (101 MHz, CHLOROFORM-d) δ 167.5, 140.2, 135.1, 133.0, 129.5, 125.9, 114.7. HRMS m/z calcd for [C_9_H_5_Cl_2_NOS + H] ^+^ 245.9469, found 245.9492.

#### 4-chloro-2-(4-chlorophenyl)isothiazol-3(2H)-one (7)

This compound (yield 28.6%) as a side product was prepared from the 3,3'-disulfanediylbis(N-(4-chlorophenyl)propanamide) **3a** (0.43 g, 1.0 mmol) and sulfuryl chloride (0.39 g, 3.0 mmol) according to the general procedure described above. ^1^H NMR (400 MHz, CHLOROFORM-d) δ 8.14 (s, 1H), 7.53–7.60 (m, *J* = 8.80 Hz, 2H), 7.41–7.48 (m, 2H); ^13^C NMR (101 MHz, CHLOROFORM-d) δ 162.2, 135.2, 133.6, 132.6, 129.7, 129.6, 125.5, 115.2. HRMS m/z calcd for [C_9_H_5_Cl_2_NOS + H]^+^ 245.9542, found 245.9540.

#### 5-chloro-2-((4-methoxyphenyl)sulfonyl)isothiazol-3(2H)-one (5b)

This compound (yield 64.2%) was prepared from the 3,3'-disulfanediylbis(N-((4-methoxyphenyl)sulfonyl)propanamide) **3b** (0.55 g, 1.0 mmol) and sulfuryl chloride (0.39 g, 3.0 mmol) according to the general procedure described above. ^1^H NMR (400 MHz, CHLOROFORM-d) δ 7.87 (d, *J* = 9.78 Hz, 2H), 7.00 (d, *J* = 8.80 Hz, 2H), 6.87 (s, 1H), 3.86 (s, 3H); ^13^C NMR (101 MHz, CHLOROFORM-d) δ 164.7, 157.9, 155.1, 131.0, 126.3, 115.4, 114.6, 77.6, 77.3, 76.9, 55.8. HRMS m/z calcd for [C10H8ClNO4S2 + Na] ^+^ 327.9475, found 327.9463.

#### 5-chloro-2-(3-chloro-4-methoxyphenyl)isothiazol-3(2H)-one (5c)

This compound (yield 57.5%) was prepared from the 3,3'-disulfanediylbis(N-(3-chloro-4-methoxybenzyl)propanamide) **3f** (0.52 g, 1.0 mmol) and sulfuryl chloride (0.39 g, 3.0 mmol) according to the general procedure described above. ^1^H NMR (400 MHz, CHLOROFORM-d) δ 7.44 (d, *J* = 2.93 Hz, 1H), 7.29 (dd, *J* = 2.45, 8.31 Hz, 1H), 7.02 (d, *J* = 8.80 Hz, 1H), 6.83 (s, 1H), 3.95 (s, 3H); ^13^C NMR (101 MHz, CHLOROFORM-d) δ 163.7, 157.4, 156.0, 129.9, 127.7, 124.9, 124.3, 123.6, 112.5, 77.4, 77.1, 76.8, 56.5. HRMS m/z calcd for [C_10_H_7_Cl_2_NO_2_S + Na] ^+^ 297.9467, found 297.9474.

#### 5-chloro-2-cyclohexylisothiazol-3(2H)-one (5d)

This compound (yield 74.5%) was prepared from the 3,3'-disulfanediylbis(N-cyclohexylpropanamide) **3g** (0.37 g, 1.0 mmol) and sulfuryl chloride (0.39 g, 3.0 mmol) according to the general procedure described above. ^1^H NMR (400 MHz, CHLOROFORM-d) δ 6.13 (s, 1H), 4.16–4.40 (m, 1H), 1.84–2.13 (m, 3H), 1.75 (d, *J* = 5.87 Hz, 2H), 1.60 (d, *J* = 13.69 Hz, 1H), 1.18–1.48 (m, 4H); ^13^C NMR (101 MHz, CHLOROFORM-d) δ 166.3, 145.5, 115.0, 77.6, 77.3, 76.9, 53.3, 33.1, 25.4, 25.0. HRMS m/z calcd for [C_9_H_12_ClNOS + H] + 218.0328, found 211.0395.

#### 5-chloro-2-(quinolin-8-ylsulfonyl)isothiazol-3(2H)-one (5e)

This compound (yield 40.6%) was prepared from the 3,3'-disulfanediylbis(N-(quinolin-8-ylsulfonyl)propanamide) **3h** (0.59 g, 1.0 mmol) and sulfuryl chloride (0.39 g, 3.0 mmol) according to the general procedure described above. ^1^H NMR (400 MHz, CHLOROFORM-d) δ 9.05–9.16 (m, 1H), 8.56 (d, *J* = 6.85 Hz, 1H), 8.29 (d, *J* = 7.82 Hz, 1H), 8.19 (d, *J* = 7.82 Hz, 1H), 7.68 (t, *J* = 7.83 Hz, 1H), 7.52–7.63 (m, 1H), 7.04 (s, 1H); ^13^C NMR (101 MHz, CHLOROFORM-d) δ 158.0, 154.7, 152.2, 143.8, 136.7, 135.7, 133.7, 129.0, 125.3, 122.7, 115.8. HRMS m/z calcd for [C_12_H_7_ClN_2_O_3_S_2_ + H] ^+^326.9587, found 326.9512.

#### 5-chloro-2-octylisothiazol-3(2H)-one (5f)

This compound (yield 42.7%) was prepared from the 3,3'-disulfanediylbis(N-octylpropanamide) **3i** (0.43 g, 1.0 mmol) and sulfuryl chloride (0.39 g, 3.0 mmol) according to the general procedure described above. ^1^H NMR (400 MHz, CHLOROFORM-d) δ 6.20 (s, 1H), 3.67 (t, *J* = 7.34 Hz, 2H), 1.53–1.78 (m, 2H), 1.17—1.32 (m, 10H), 0.72–0.95 (m, 3H); ^13^C NMR (101 MHz, CHLOROFORM-d) δ 166.8, 145.4, 114.7, 43.7, 31.7, 29.6, 29.0, 29.0, 26.4, 22.6, 14.0. HRMS m/z calcd for [C_11_H_18_ClNOS + H] ^+^ 248.0870, found 248.0872.

#### 5-chloro-2-(prop-2-yn-1-yl)isothiazol-3(2H)-one (5 g)

This compound (yield 46.1%) was prepared from the 3,3'-disulfanediylbis(N-octylpropanamide) **3h** (0.43 g, 1.0 mmol) and sulfuryl chloride (0.39 g, 3.0 mmol) according to the general procedure described above. ^1^H NMR (400 MHz, CHLOROFORM-d) δ 6.25 (s, 1H), 4.51 (d, *J* = 1.96 Hz, 3H), 2.49 (t, *J* = 2.45 Hz, 1H); ^13^C NMR (101 MHz, CHLOROFORM-d) δ 166.3, 147.2, 114.2, 76.6, 75.3, 33.0. HRMS m/z calcd for [C_6_H_4_ClNOS + H] ^+^173.9702, found 173.9756.

#### 4,5-dichloro-2-(4-chlorophenyl)isothiazol-3(2H)-one (6)

To a well-stirred solution of the 3,3'-disulfanediylbis(N-(4-chlorophenyl)propanamide) **3a** (0.43 g, 1.0 mmol) in DCM (25 mL) was added 5.0 equiv. of SO_2_Cl_2_ (0.65 g, 5.0 mmol) in an ice bath. After 2 h, the solution was poured into water and extracted with DCM. The organic layers were dried over anhydrous MgSO_4_, filtered and evaporated under reduced pressure. The resulting residue was purified by flash column chromatography on silica gel to afford the desired compound **6a**. ^1^H NMR (400 MHz, CHLOROFORM-d) δ 7.48–7.58 (m, 3H), 7.38–7.48 (m, 2H); ^13^C NMR (101 MHz, CHLOROFORM-d) δ 160.4, 139.3, 134.4, 133.9, 129.9, 125.7, 115.3. HRMS m/z calcd for [C_9_H_4_Cl_3_NOS + H]^+^ 279.9152, found 279.9151.

#### 4-bromo-2-(4-chlorophenyl)isothiazol-3(2H)-one (8)

To a well-stirred solution of the 2-(4-chlorophenyl)isothiazol-3(2H)-one **4a** (0.21 g, 1.0 mmol) in DCM (25 mL) was added 1.0 equiv. of Br_2_ (0.13 g, 1.0 mmol) in an ice bath. After 2 h, the solution was poured into water and extracted with DCM. The organic layers were dried over anhydrous MgSO_4_, filtered and evaporated under reduced pressure. The resulting residue was purified by flash column chromatography on silica gel to afford the desired compound **8**. ^1^H NMR (400 MHz, CHLOROFORM-d) δ 8.25 (s, 1H), 7.56 (d, *J* = 7.82 Hz, 2H), 7.40–7.51 (m, 2H); ^13^C NMR (101 MHz, CHLOROFORM-d) δ 162.9, 135.3, 135.2, 133.6, 129.7, 125.5, 102.6. HRMS m/z calcd for [C_9_H_5_BrClNOS + H] ^+^ 289.9037, found 289.9047.

#### 5-chloro-2-(4-chlorophenyl)isothiazol-3(2H)-one 1-oxide (9)

To a well-stirred solution of the 5-chloro-2-(4-chlorophenyl)isothiazol-3(2H)-one **5a** (0.25 g, 1.0 mmol) in DCM (25 mL) was added dropwise 3-chloroperoxybenzoic acid (0.21 g, 1.2 mmol) in DCM in an ice bath. After 3 h, the solution was poured into water and extracted with DCM. The organic layers were dried over anhydrous MgSO_4_, filtered and evaporated under reduced pressure. The resulting residue was purified by flash column chromatography on silica gel to afford the desired compound **9**. ^1^H NMR (400 MHz, CHLOROFORM-d) δ 7.44–7.51 (m, 2H), 7.34–7.41 (m, 2H), 6.85 (s, 1H); ^13^C NMR (101 MHz, CHLOROFORM-d) δ 163.5, 157.3, 135.3, 131.1, 130.1, 128.7, 124.3, 77.4, 77.1, 76.8. HRMS m/z calcd for [C_9_H_5_Cl_2_NO_2_S + H] ^+^261.9418, found 261.9408.

#### Antimicrobial susceptibility testing

MRM-resistant *E. coli* BL21 (NDM-1) carrying only an IPTG-inducible plasmid pET28b-bla NDM-1 was produced from a parental *E. coli* BL21 strain without producing NDM-1 for MIC determination of tested compounds. *E. coli* HN88 carrying blaNDM-1 was isolated from urine specimens of urinary tract infected patient. CRE strains shown in Table 3 were collected from the patients’ urine, feces, and sputum in the Second People’s Hospital of Jiaxing in Zhejiang Province, China. It should be noted that humans were not involved in the current study. The aforementioned isolated strains were provided by the hospital. Strains including *E. coli* and *S. aureus* in Table 2 were purchased from the American Type Culture Collection (ATCC, Manassas, VA).

#### MIC determination

A collection of *E. coli* BL21 (NDM-1) were incubated overnight on the Mueller–Hinton agar (MHA) plate at 37 °C under aerobic conditions followed by transferring to normal saline (NS) where the OD600 value of the NS solution ranged from 0.08 to 0.1. The NS solution consisting of *E. coli* BL21 (NDM-1) was then transferred again to a 96-well plate and incubated with Mueller–Hinton broth (MHB), 1 mM of IPTG, and a serial concentration of MRM alone, the freshly prepared compound alone in DMSO, or a combination of both at the ratio 1 to 1. After being incubated at 37 °C overnight, the MIC values of the 16 antimicrobial agents were determined using a broth microdilution method following CLSI guidelines and our previous study. [7, 19, 20]

#### Cytotoxicity (IC_50_) test of compound 5a towards the L929 Cell Line

According to the method reported before, the standard 3-(4,5-dimethylthiazol-2-yl)-5(3-carboxymethonyphenol)-2-(4-sulfophenyl)-2H-tetrazolium (MTS) assay was performed to evaluate the cytotoxicity of most potent compound **5a** against the mouse peritoneal fibroblast L929 cells [19, 20, 29]. The half-maximal inhibitory concentration (IC_50_) of compound **5a** was measured by using a Cell Titer 96 AQueous assay. Briefly, L929 Cells at a density of 10,000 cells were exposed to various concentrations compound **5a** in a final volume of 100 μL in each well of a 96-well plate, followed by 48 h incubation at 37 °C. The negative group was comprised of a cell mixture containing 0.1% DMSO and the blank group consisted of the DMEM (10% FBS) medium alone without cells. The medium was removed from the plates after treatment with compound **5a**, followed by addition of the freshly prepared MTS/phenazine methosulfate mixture at a ratio of 20:1 in PBS and DMEM into each well and further incubation for 2 h at 37 °C. The optical density was determined at 490 nm by a Microplate Reader (Clariostar, BMG). Percentage cell survival was calculated as follows: (corrected reading from the test well–corrected reading from the blank well)/ (corrected reading from negative well–corrected reading from the blank well) × 100%. The IC_50_ value of compound **5a** was estimated from the dose−response curve of the MTS assay by using software GraphPad Prism 9. All experiments were performed in triplicates.

#### Growth curve of compound 5a against *S. aureus* 43,300

An inoculum size of Gram-positive strain *S. aureus* **43,300** was incubated at 37 ºC in the presence of compound **5a** at the concentrations equal to 1/4 MIC, 1/2 MIC, MIC, twice the MIC, and four times the MIC for overnight. A control test was performed for the organisms without compound **5a**. Aliquots of 1.0 mL of the samples were collected at time intervals of 0, 4, 6, 8, 12, and 22 h and inoculated aseptically with subjection to a series of tenfold dilution on brain–heart infusion (BHI) broth plates followed by incubating at 37 ºC for 24 h. Their growth was checked by measuring the absorbance at 600 nm. The procedure was performed in triplicate and a graph of the CD_600_ against time was plotted.

#### Time-dependent killing assay

The bacterial suspensions of MRSA 43300 were adjusted in Luria–Bertani broth (LB) to 10^6^ CFU/mL, followed by treatment with the above-mentioned concentrations of compound **5a** at 37 °C with continuous shaking (200 rpm). Viable bacterial cells at each time point (0, 4, 8, 12, and 22 h) were counted. The procedure was performed in triplicate and the time-killing curve were plotted using GraphPad 8.0 (San Diego, CA, USA).

#### Evaluation of the in vivo antibiotic activity using a BALB/c mice model of MRSA infection

To evaluate the in vivo bactericidal efficacy of compound **5a**, a MRSA infection model of BALB/c mice was employed as previously described with little modification [7, 30–32]. MRSA infection mouse was a successful whole-animal model for screening antibacterial activities of compounds. The animal study was performed in full compliance with the standard protocol approved by the animal research ethics committee of Yunnan University of Chinese Medicine. Six-week-old BALB/c mice were purchased from the Guangdong Center for Experimental Animals. All BALB/c mice were kept in a constant temperature at 22 °C and 60% relative humidity with a period of 12 h light–dark cycle and given free access to standard diet and water. Briefly, the cultures of MRSA ATCC 43300 grown overnight at 37 °C in BHI broth were diluted 1:100 using fresh TSB medium and incubated in an incubator shaker for 3 h. Log phase cells were pelleted and washed twice with sterile phosphate buffered saline (PBS) before being resuspended in 100 mL of PBS for further use. Using a Hamilton syringe, BALB/c mice (N = 10) were treated via the lateral tail-vein injection with MRSA ATCC 43300 suspended in PBS at a dose of 10^9^ CFU. A solution of compound **5a** in the formulation of 5% Cremophor EL, 5% ethanol and 90% saline was freshly prepared at a concentration of 2 mg/mL on the day of use and used for animal study within 0.5 h. Various treatments including vehicle consisting of 5% CremophorEL, 5% ethanol and 90% saline, compound **5a** alone at a concentration of 0.5 mg/kg were administered intraperitoneal injections (IP) per 12 h post infection respectively. The Survival rate of BALB/c mice was recorded at 12 h interval for 4 days after MRSA ATCC 43300 challenge. BALB/c mice were considered dead when they were immobile and no longer responding to physical stimuli. Experimental survival animals will be killed by cervical dislocation to ensure immediate death and not cause unnecessary/prolonged pain to them in accordance with ARRIVE guidelines. Data were analyzed for statistical significance using a log-rank and χ square test with 1 degree of freedom.

### Supplementary Information

**Additional file 1: Figure S1.** ^1^H and ^13^C NMR spectra of **2-(4-chlorophenyl)isothiazol-3(2H)-one (4a). Figure S2.** ^1^H and ^13^C NMR spectra of **2-((4-methoxyphenyl)sulfonyl)isothiazol-3(2H)-one (4b). Figure S3.** ^1^H and ^13^C NMR spectra of **2-(4-bromobenzyl)isothiazol-3(2H)-one (4c). Figure S4.** ^1^H and ^13^C NMR spectra of **5-chloro-2-(4-chlorophenyl)isothiazol-3(2H)-one (5a).** ^13^C NMR (101 MHz, CHLOROFORM-d) 167.5, 140.2, 135.1, 133.0, 129.5, 125.9, 114.7. **Figure S5.** ^1^H and ^13^C NMR spectra of **5-chloro-2-((4-methoxyphenyl)sulfonyl)isothiazol-3(2H)-one (5b). Figure S6.** ^1^H and ^13^C NMR spectra of **5-chloro-2-(3-chloro-4-methoxyphenyl)isothiazol-3(2H)-one (5c). Figure S7.** ^1^H and ^13^C NMR spectra of **5-chloro-2-cyclohexylisothiazol-3(2H)-one (5d). Figure S8.** ^1^H and ^13^C NMR spectra of **5-chloro-2-(quinolin-8-ylsulfonyl)isothiazol-3(2H)-one (5e). Figure S9.** ^1^H and ^13^C NMR spectra of **5-chloro-2-octylisothiazol-3(2H)-one (5f). Figure S10.** ^1^H and ^13^C NMR spectra of **5-chloro-2-(prop-2-yn-1-yl)isothiazol-3(2H)-one (5g). Figure S11.** ^1^H and ^13^C NMR spectra of **4,5-dichloro-2-(4-chlorophenyl)isothiazol-3(2H)-one (6). Figure S12.** ^1^H and ^13^C NMR spectra of **4-chloro-2-(4-chlorophenyl)isothiazol-3(2H)-one (7). Figure S13.** ^1^H and ^13^C NMR spectra of **4-bromo-2-(4-chlorophenyl)isothiazol-3(2H)-one (8). Figure S14.** ^1^H and.^13^C NMR spectra of **5-chloro-2-(4-chlorophenyl)isothiazol-3(2H)-one 1-oxide (9)**.

## Data Availability

The datasets used and/or analyzed during the current study are available from the corresponding author on reasonable request.
