# Supplementary material for: Identification of isothiazolones analogues as potent bactericidal agents against antibiotic resistant CRE and MRSA strains
Source: BMC Chem. 2023 Dec 16;17(1):183. doi: 10.1186/s13065-023-01100-3 (PMC10724953; doi:10.1186/s13065-023-01100-3)
Supplement: Supplementary file 1 — Additional file 1: Figure S1. 1H and 13C NMR spectra of 2-(4-chlorophenyl)isothiazol-3(2H)-one (4a). Figure S2. 1H and 13C NMR spectra of 2-((4-methoxyphenyl)sulfonyl)isothiazol-3(2H)-one (4b). Figure S3. 1H and 13C NMR spectra of 2-(4-bromobenzyl)isothiazol-3(2H)-one (4c). Figure S4. 1H and 13C NMR spectra of 5-chloro-2-(4-chlorophenyl)isothiazol-3(2H)-one (5a). 13C NMR (101 MHz, CHLOROFORM-d) 167.5, 140.2, 135.1, 133.0, 129.5, 125.9, 114.7. Figure S5. 1H and 13C NMR spectra of 5-chloro-2-((4-methoxyphenyl)sulfonyl)isothiazol-3(2H)-one (5b). Figure S6. 1H and 13C NMR spectra of 5-chloro-2-(3-chloro-4-methoxyphenyl)isothiazol-3(2H)-one (5c). Figure S7. 1H and 13C NMR spectra of 5-chloro-2-cyclohexylisothiazol-3(2H)-one (5d). Figure S8. 1H and 13C NMR spectra of 5-chloro-2-(quinolin-8-ylsulfonyl)isothiazol-3(2H)-one (5e). Figure S9. 1H and 13C NMR spectra of 5-chloro-2-octylisothiazol-3(2H)-one (5f). Figure S10. 1H and 13C NMR spectra of 5-chloro-2-(prop-2-yn-1-yl)isothiazol-3(2H)-one (5g). Figure S11. 1H and 13C NMR spectra of 4,5-dichloro-2-(4-chlorophenyl)isothiazol-3(2H)-one (6). Figure S12. 1H and 13C NMR spectra of 4-chloro-2-(4-chlorophenyl)isothiazol-3(2H)-one (7). Figure S13. 1H and 13C NMR spectra of 4-bromo-2-(4-chlorophenyl)isothiazol-3(2H)-one (8). Figure S14. 1H and.13C NMR spectra of 5-chloro-2-(4-chlorophenyl)isothiazol-3(2H)-one 1-oxide (9). [file 13065_2023_1100_MOESM1_ESM.docx]

Additional file for

**Identification of Isothiazolones Analogues as Potent Bactericidal Agents against Antibiotic Resistant CRE and MRSA Strains**

Wenbin Jin,*^‡,1,2^ Chen Xu,^‡,2,3^ Ning Dong,^2,5^ Kaichao Chen, ^2^ Die Zhang,^1^ Jinhua Ning,^1^ Yunbing Li,^5^ Guangfen Zhang,^5^ Jin Ke,^1^ Anguo Hou,^1^ Linyun Chen,^1^ Sheng Chen,* ^4^ and Kin-Fai Chan*^2^

*^1^* Key Laboratory of External Drug Delivery System and Preparation Technology in Universities of Yunnan and Faculty of Chinese Materia Medica, Yunnan University of Chinese Medicine, Kunming, Yunnan, China

*^2^* State Key Laboratory of Chemical Biology and Drug Discovery and Department of Applied Biology and Chemical Technology, The Hong Kong Polytechnic University, Hung Hom, Kowloon, Hong Kong SAR, China

*^3^* School of Medicine, Jiangsu University, Zhenjiang, Jiangsu, China

^4^ Department of Food Science and Nutrition, The Hong Kong Polytechnic University, Hung Hom, Kowloon, Hong Kong SAR, China

^5^Department of Medical Microbiology, School of Biology and Basic Medical Sciences, Suzhou Medical College of Soochow University, Suzhou, China

*^‡^*These authors contributed equally.

*Corresponding authors: Wenbin Jin, Sheng Chen and Kin-Fai Chan

For W. B. J, email: 421810873@qq.com

For S. C., email: sheng.chen@polyu.edu.hk

For K. F. C., email: kf.chan@polyu.edu.hk

**Table of Content**

| **Additional file 1: Figure S1–S14** | ^1^H and ^13^C spectra of compounds **4a-4c**, **5a-5g, and 6-9.** | Page S3 – S13 |
| --- | --- | --- |
|  |  |  |
|  |  |  |

**Figure S1.** ^1^H and ^13^C NMR spectra of **2-(4-chlorophenyl)isothiazol-3(2H)-one (4a)**

**Figure S2.** ^1^H and ^13^C NMR spectra of **2-((4-methoxyphenyl)sulfonyl)isothiazol-3(2H)-one (4b)**

**Figure S3.** ^1^H and ^13^C NMR spectra of **2-(4-bromobenzyl)isothiazol-3(2H)-one (4c)**

**Figure S4.** ^1^H and ^13^C NMR spectra of **5-chloro-2-(4-chlorophenyl)isothiazol-3(2H)-one (5a)**

^13^C NMR (101 MHz, CHLOROFORM-d)  167.5, 140.2, 135.1, 133.0, 129.5, 125.9, 114.7

**Figure S5.** ^1^H and ^13^C NMR spectra of **5-chloro-2-((4-methoxyphenyl)sulfonyl)isothiazol-3(2H)-one (5b)**

^^


**Figure S6.** ^1^H and ^13^C NMR spectra of **5-chloro-2-(3-chloro-4-methoxyphenyl)isothiazol-3(2H)-one (5c)**

**Figure S7.** ^1^H and ^13^C NMR spectra of **5-chloro-2-cyclohexylisothiazol-3(2H)-one (5d)**

**Figure S8.** ^1^H and ^13^C NMR spectra of **5-chloro-2-(quinolin-8-ylsulfonyl)isothiazol-3(2H)-one (5e)**

**Figure S9.** ^1^H and ^13^C NMR spectra of **5-chloro-2-octylisothiazol-3(2H)-one (5f)**

**Figure S10.** ^1^H and ^13^C NMR spectra of **5-chloro-2-(prop-2-yn-1-yl)isothiazol-3(2H)-one (5g)**

**Figure S11.** ^1^H and ^13^C NMR spectra of **4,5-dichloro-2-(4-chlorophenyl)isothiazol-3(2H)-one (6)**

**Figure S12.** ^1^H and ^13^C NMR spectra of **4-chloro-2-(4-chlorophenyl)isothiazol-3(2H)-one (7)**

**Figure S13.** ^1^H and ^13^C NMR spectra of **4-bromo-2-(4-chlorophenyl)isothiazol-3(2H)-one (8)**

**Figure S14.** ^1^H and ^13^C NMR spectra of **5-chloro-2-(4-chlorophenyl)isothiazol-3(2H)-one 1-oxide (9)**
